# Histopathological and microbiological study of porcine lymphadenitis: contributions to diagnosis and control of the disease

**DOI:** 10.1186/s40813-020-00172-0

**Published:** 2020-12-04

**Authors:** Fernando Cardoso-Toset, Jaime Gómez-Laguna, Lidia Gómez-Gascón, Irene M. Rodríguez-Gómez, Angela Galán-Relaño, Librado Carrasco, Carmen Tarradas, Ana I. Vela, Inmaculada Luque

**Affiliations:** 1 CICAP – Food Research Centre, Pozoblanco, 14400 Córdoba, Spain; 2 Department of Anatomy and Comparative Pathology, University of Córdoba, International Excellence Agrifood Campus ‘CeiA3’, 14071 Córdoba, Spain; 3 Department of Animal Health, University of Córdoba, International Excellence Agrifood Campus ‘CeiA3’, 14071 Córdoba, Spain; 4 Department of Animal Health, Faculty of Veterinary Medicine, Complutense University, Madrid, Spain; 5 VISAVET Health Surveillance Centre, Faculty of Veterinary Medicine, Complutense University, Madrid, Spain

**Keywords:** Free-range pigs, Lymphadenitis, Tuberculosis like lesions, *Mycobacterium tuberculosis complex*, *Trueperella pyogenes*, *Streptococcus* spp.

## Abstract

Tuberculosis like lesions (TBL) in free-range pigs are characterised by presenting a marked heterogeneity in pathology and microbiology features, with a notorious role of *Mycobacterium tuberculosis* complex (MTC), *Trueperella pyogenes* and different *Streptococcus* species. However, the capacity of these microorganism to spread to different organic cavities leading to a generalised disease is unknown. Therefore, this study evaluated the organic distribution of these agents in free-range pig carcasses whole condemned due to generalised TBL.

A total of 37 totally condemned animals were analysed, and samples of lymph nodes and organs were obtained (*n* = 262) and subjected to histopathological and microbiological examination. In addition, *T. pyogenes* and streptococci species were further characterised by PFGE analysis. Two different patterns were evidenced with lack or occasional lesions in superficial inguinal (SILN) and popliteal (PLN) lymph nodes and advanced lesions in submandibular (SLN) (35/36) and gastrohepatic (GHLN) (33/35) lymph nodes (stages III and IV). Early stage granulomas (stage I and II) prevailed in lungs (16/20), liver (14/31) and spleen (7/18). The microbiological analysis revealed that MTC, detected by qPCR, was present in 31 out of 37 animals and 90 (90/262) samples. In 26 out of the 31 pigs, MTC was detected from two or more organs. SLN (24/31) and GHLN (19/31) were the MTC^+^ organs most frequently detected, with 29 out of 31 MTC^+^ pigs detected as positive in one or both samples, which points out that both lymph nodes must be included in the sampling of surveillance programs. Other pathogens, such as *T. pyogenes* and *Streptococcus* spp., were also involved in generalised lymphadenitis, being frequently isolated from SLN and other organs, such as liver (*T. pyogenes*), tonsils or lung (*Streptococcus* spp.). A wide genetic diversity among streptococci was observed, showing the ubiquitous character of these pathogens, however, the isolation of a single clone of *T. pyogenes* from different organic locations from animals with generalised TBL was a common finding of this study, highlighting that the role of this pathogen in porcine lymphadenitis may be underestimated. These results should be considered in future studies on the pathogenesis and control of porcine lymphadenitis.

## Background

Porcine lymphadenitis is commonly an asymptomatic disease that involves the inflammation of superficial and deep lymph nodes in response to infection by different microorganisms, which can spread to other organs, mainly lungs, liver and spleen [15, 17]. Macroscopically, these lesions are characterised by their nodular appearance, which may be caseous, purulent or proliferative [4, 10] and are frequently listed in the literature as tuberculosis-like lesions (TBL). TBL are most detected during *postmortem* inspection and result in partial or whole carcass condemnation at the slaughterhouse with a relevant economic impact for producers [15].

TBL are characterised by presenting a marked heterogeneity in pathology, microbiology, and immunological features both in humans and animals [5, 7, 12]. In this sense, not only different lesional patterns may be identified by histopathology but also a wide range of microorganisms can be detected, with mycobacteria belonging to *Mycobacterium avium* complex (MAC), *Mycobacterium tuberculosis* complex (MTC) and *Rhodococcus equi* as the species most frequently associated with TBL in domestic and feral pigs [2, 8, 16, 17, 21, 24, 25]. However, the complex aetiology and wide spectrum of microorganisms different to mycobacteria that can be involved in TBL in free-range pigs have been recently evidenced, with *Trueperella pyogenes* and several *Streptococcus* species underscored as the main non-tuberculous microorganisms associated with these lesions [7].

According to the chronic course of this disease, the identification of the causative agents can be a complicated task, especially when several pathogens or different isolates of the same species may be involved. These facts may be important in establishing the diagnostic methods and the control measures [5, 7, 17]. The characteristics and distribution of the lesions and bacteria identified provide valuable information on the mechanisms of transmission and the role played by the pig in the maintenance and dissemination of diseases of importance to both animal and public health [23, 29]. This information will allow gaining knowledge of interest to understand the pathogenesis of TBL in pigs as well as identifying target organs for the diagnosis of the main pathogens involved in this process to avoid misdiagnosis and the implementation of holistic control measures. Therefore, the organic distribution of MTC, *T. pyogenes* and *Streptococcus* species involved in TBL from whole condemned free-ranged pig carcasses due to generalised lymphadenitis was evaluated in this study. In a second step, non-tuberculous microorganisms were further characterised to determine the genetic similarity among isolates obtained from different organs from the same carcass with generalised disease.

## Results

### Histopathological analysis

A total of 206 samples belonging to 37 animals were subjected to the histopathological examination. Since samples needed to be split into two portions, all samples were not always available to perform all the studies.

**Fig. 1:**
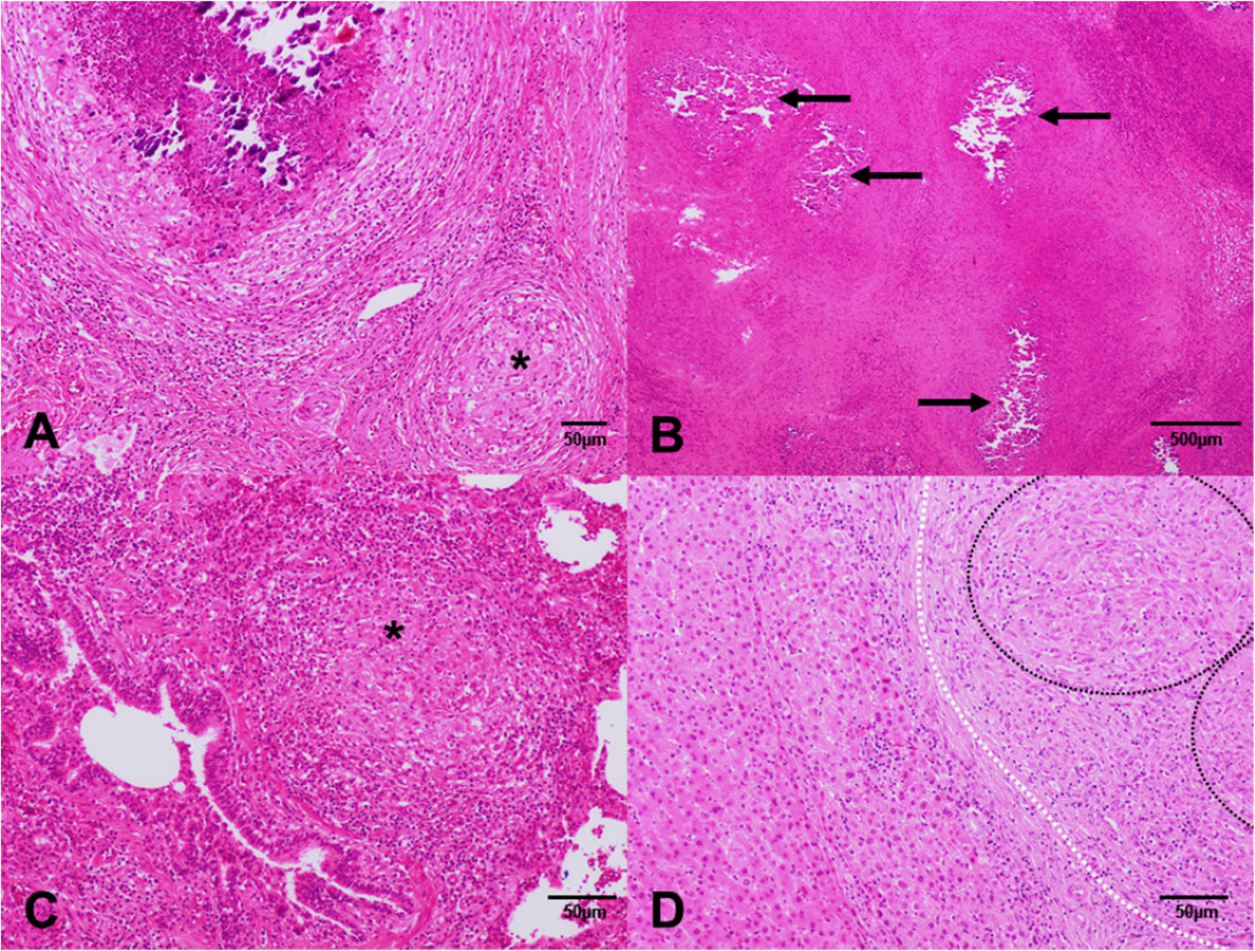
Granuloma stages (I-IV) in tissues from pigs. **a** Granuloma, submandibular lymph node, pig. Stage IV granuloma with a thick connective tissue capsule, and a prominent caseous necrotic core with multifocal islands of mineralization, accompanied by a satellite stage II granuloma (asterisk), composed by epithelioid macrophages enclosed by a thin capsule, with peripheral infiltration of scattered lymphocytes. Hematoxylin and eosin (HE). Bar, 50 μm. **b** Granuloma, submandibular lymph node, pig. Multicentric granulomas with several mineralization foci (arrows) as well as extensive necrosis. HE. Bar, 500 μm. **c** Granuloma, lung, pig. Clustered epithelioid macrophages surrounded by lymphocytes and erythrocytes in a stage I granuloma in the lung. HE. Bar, 50 μm. **d** Granuloma, liver, pig. Coalescent stage II granulomas (dashed black circles) showing epithelioid macrophages enclosed by a thin connective tissue capsule (dashed white line), with mild peripheral infiltration of scattered lymphocytes. HE. Bar, 50 μm

**Fig. 2:**
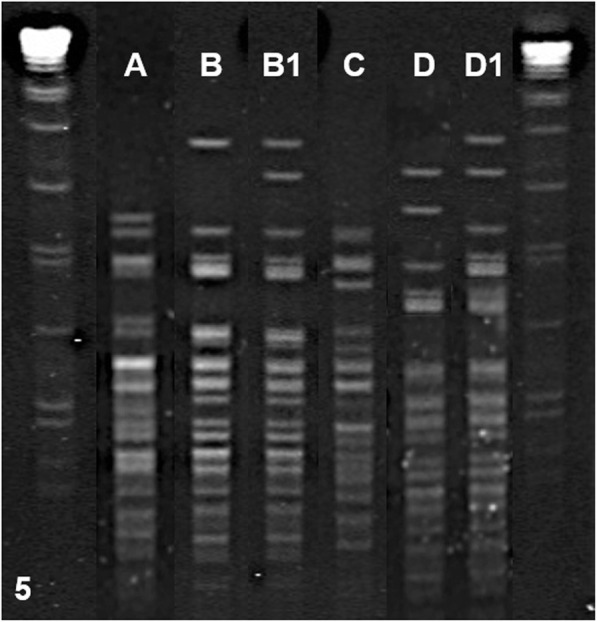
Molecular characterisation of *Trueperella pyogenes* isolates by PFGE, using the restriction enzyme *Bcu*I. A pattern of 15 to 18 well differentiated bands in the gel were obtained, grouping the isolates in 6 different pulsotypes (A, B, B1, C, D and D1)

Two different lesional patterns were evidenced in lymph nodes with lack or occasional lesions observed in SILN and PLN and prominent and advanced lesions detected in SLN and GHLN. Therefore, only 1 out of 18 SILN and 4 out of 34 PLN presented granulomatous inflammation consisting of a combination of granulomas of different stages. Late stage granulomas (stages III and IV) were overrepresented in SLN and GHLN (35/36 and 33/35 samples, respectively), which were commonly found in combination with satellite pyogranulomas and granulomas of earlier stages (stages I and II) (Fig. 1). Numerous multicentric granulomas with several mineralisation foci as well as extensive necrosis were frequent (Fig. 2). Stage IV granulomas was the only lesion found in three SLNs and two GHLNs. No microscopic lesions were observed in 1/36 SLN and 2/35 GHLN.

Early stage granulomas (stage I and II) were the predominant lesions observed in lungs (16/20) (Fig. 3), liver (14/31) (Fig. 4) and spleen (7/18), which were usually detected in combination with pyogranulomas and granulomas of later stages (stage III and IV granulomas, but usually with only one or two multicentric granulomas). Numerous pyogranulomas were uniquely found in the lung of one animal. No microscopic lesions were observed in 1/20 lung, 9/31 liver and 7/18 spleen samples, despite they were grossly evidenced (data not shown).

### TB diagnosis

MTC was detected (MTC^+^) in 31 (83.78%) out of 37 animals and 90 (34.35%) out of 262 samples, respectively (Table 1). Considering the organic location, SLN (24/31; 77.42%) and GHLN (19/31; 61.29%) were the organs where MTC was most frequently detected by qPCR, with 29 (93.55%) out of 31 MTC^+^ animals yielding a positive result in at least one of these lymph nodes.

**Table 1:** Organic distribution of isolated microorganisms in 37 pigs with generalised TBL

Microorganisms	Positive animals	Positive samples	Organic distribution of detected microorganisms*							
			SLN	PLN	SILN	GHLN	Lungs	Liver	Spleen	Tonsils
MTC	31 (83.78)	90 (34.35)	**24 (64.86)**	7 (19.44)	11 (30.55)	**19 (52.78)**	8 (21.62)	8 (23.53)	9 (37.50)	4 (19.05)
*T. pyogenes*	16 (43.24)	40 (26.31)	7 (18.92)	5 (13.89)	7 (19.44)	3 (8.33)	5 (13.51)	**8 (23.53)**	3 (12.5)	2 (9.52)
*S. porcinus*	15 (40.54)	28 (18.42)	**4 (10.81)**	0 (0)	3 (8.33)	2 (5.55)	**5 (13.51)**	2 (5.88)	3 (12.5)	**9 (42.86)**
*S. dysgalactiae spp equisimilis*	14 (37.84)	25 (16.45)	**5 (13.51)**	0 (0)	4 (11.11)	4 (11.11)	**5 (13.51)**	3 (8.82)	2 (8.33)	2 (9.52)
*S. suis*	8 (18.92)	8 (4.60)	1 (2.70)	0 (0)	1 (2.78)	1 (2.78)	2 (5.55)	0 (0)	0 (0)	2 (9.52)
*Aerococcus spp*^a^	7 (18.92)	9 (5.92)	1 (2.70)	2 (5.55)	3 (8.33)	1 (2.78)	0 (0)	0 (0)	0 (0)	2 (9.52)
*Corynebacterium spp*^b^	4 (10.81)	9 (5.92)	2 (5.40)	2 (5.55)	1 (2.78)	0 (0)	2 (5.55)	2 (5.88)	0 (0)	0 (0)
*S. equi zooepidemicus*	4 (10.81)	6 (3.95)	0 (0)	0 (0)	0 (0)	2 (5.55)	1 (2.70)	0 (0)	1 (4.17)	2 (9.52)
*S. dysgalactiae* spp. *dysgalactiae*	2 (5.40)	4 (2.63)	1 (2.70)	0 (0)	2 (5.55)	0 (0)	1 (2.70)	0 (0)	0 (0)	0 (0)
*S. agalactiae*	3 (8.11)	3 (1.97)	1 (2.70)	0 (0)	0 (0)	0 (0)	0 (0)	1 (2.94)	0 (0)	1 (4.76)
*Rhodococcus equi*	1 (2.70)	3 (1.97)	1 (2.70)	0 (0)	0 (0)	0 (0)	1 (2.70)	1 (2.94)	0 (0)	0 (0)
*Globicatella sanguinis*	1 (2.70)	3 (1.97)	0 (0)	0 (0)	0 (0)	0 (0)	1 (2.70)	1 (2.94)	1 (4.17)	0 (0)
*S. mitis*	3 (8.11)	3 (1.97)	0 (0)	2 (5.55)	0 (0)	0 (0)	0 (0)	1 (2.94)	0 (0)	0 (0)
*S. equinus*	2 (5.40)	2 (1.31)	1 (2.70)	0 (0)	0 (0)	0 (0)	0 (0)	0 (0)	1 (4.17)	0 (0)
*Streptococcus bovis*	2 (5.40)	2 (1.31)	2 (5.40)	0 (0)	0 (0)	0 (0)	0 (0)	0 (0)	0 (0)	0 (0)
Other streptococci^c^	5 (13.51)	5 (3.29)	0 (0)	2 (5.55)	0 (0)	1 (2.78)	1 (2.70)	1 (2.94)	0 (0)	0 (0)
Other microorganisms^d^	4 (8.11)	3 (1.97)	3 (8.11)	0 (0)	0 (0)	0 (0)	0 (0)	1 (2.94)	0 (0)	0 (0)
TOTAL	37 (100)	262 (100)	37	36	36	36	37	34	24	21

*SLN, PLN, SILN and GHLN: submandibular, popliteal, superficial inguinal and gastrohepatic lymph nodes, respectively

^a^*A. viridans* (8 isolates, 6 positive animals), *A.urinae* (1 isolate)

^b^*C. striatum/amynocolatum* (6 isolates, 2 positive animals), *C. urealitycum* (3 isolates, 2 positive animals)

^c^*S. uberis, S. salivarius, S. oralis* (1 isolate each), *Streptococcus* spp. (2 isolates, 2 positive animals)

^d^*Mycobacterium avium* complex*, Cellulomonas/Microbacterium, Lactococcus lactis* and *Erysipelothrix rhusiopatiae* (1 isolate each)

MTC was also detected from spleen (9/31), SILN (11/31), lungs (8/31), liver (8/31) and tonsils (4/31) from MTC^+^ animals (Table 1). MAC was only detected in one pig with TBL in the liver. In 26 (83.87%) out of the 31 MTC^+^ animals this pathogen was detected in two or more organic locations.

A predominance of mineralised lesions (stage IV granulomas) were observed in lymph nodes of the six MTC negative (MTC^−^) animals. However, in 4 out of these 6 MTC^−^ animals early stage granulomas (stage I and stage II granulomas) were also observed either in lymph nodes or in the other examined organs, such as lungs, liver or spleen.

### Bacterial isolation

A total of 152 isolates were obtained (Table 1) with *T. pyogenes*, *S. porcinus* and *S. dysgalactiae* spp. *equisimilis* as the most frequently non-tuberculous microorganisms detected from animals (43.24, 40.54, 37.84%, respectively) and analysed samples (26.31, 18.42, 16.45%, respectively). A wide tissue distribution was observed for these microorganisms, with emphasis mainly on *T. pyogenes* isolation from the liver (8/34; 23.53%), SLN (7/37; 18.92%) and SILN (7/36; 19.44%); *S. porcinus* detection in tonsils (9/21; 42.86%), lungs (5/37; 13.51%) and SLN (4/37; 10.81%); and *S. dysgalactiae* spp. *equisimilis* in SLN (5/37; 13.51%) and lungs (5/37; 13.51%). Other bacteria isolated with lower frequency are showed in Table 1.

Regarding bacterial dissemination, *T. pyogenes* was isolated from two or more organs in 8/16 (50.0%) animals, *S. porcinus* in 5/15 (33.33%) and *S. dysgalactiae* spp. *equisimilis* in 6/14 (42.86%) animals, respectively (Table 2). These isolates were further analysed by PFGE analysis.

**Table 2:** PFGE analysis of selected microorganisms isolated from different organic localization from pigs with generalised TBL

Microorganism	Animal ID	PFGE patterns identified								Farm
		SLN^a^	PLN	SILN	GHLN	Lungs	Liver	Spleen	Tonsils	
*T. pyogenes*	#6	-^*^	–	–	-^*^	**A**	A^*^	–	–	5
	#8	A^*^	**A**	**A**	**A**	**A**	**A**	–	–	6
	#11	-^*^	–	**A**	-^*^	–	A^*^	**A**	–	7
	#19	-^*^	-^*^	**B**	–	–	**B**	-^*^	**B**	7
	#20	-^*^	C	B	-^*^	B	B	–	–	7
	#21	C^*^	-^*^	D	–	C	C^*^	–	C	12
	#22	D1^*^	D	B1	B1	B	B1	B	–	12
	#23	–	B^*^	B^*^	-^*^	-^*^	-^*^	-^*^	-^*^	12
*S. porcinus*	#10^*^	G	**–**	H	–	–	–	I	–	7
	#19^*^	–	**–**	C	–	–	A	–	C	12
	#23^*^	–	**–**	–	–	B	–	–	C	12
	#26^*^	E	**–**	–	F	E	F	F	E	14
	#34	–	**–**	**D**	**D**	**D**	**–**	**–**	**D**	11
*S. dysgalactiae* spp. *equisimilis*	#7^*^	–	**–**	B	A	A	–	–	–	6
	#9^*^	C	**–**	–	D	C	C	–	–	7
	#29^*^	–	**–**	–	–	**F**	**F**	–	–	15
	#33	–	**–**	G	–	F	–	–	G1	15
	#35^*^	–	**–**	–	–	F	F	E	–	16
	#37^*^	–	**–**	**H**	**H**	–	–	–	–	4

^a^SLN, PLN, SILN and GHLN: submandibular, popliteal, superficial inguinal and gastrohepatic lymph nodes, respectively

Different letters (A, B-B1, C, D-D1) correspond with different PFGE patterns

-: negative

^*^MTC detected from this sample by qPCR analysis

Cases with the same PFGE pattern isolated from two or more organs are marked in bold

### PFGE analysis

Thirty-two *T. pyogenes* isolates belonging to eight animals with systemic dissemination of the bacterium were selected to be further characterised by PFGE. Four different PFPs (A, B-B1, C, D-D1) were identified at an 85% of genetic similarity after *Bcu*I DNA (4/32, GD 0.13) digestion (Table 2, Fig. 5). In 5 animals (5/8; 62.5%) all isolates displayed an undistinguishable PFGE macrocrestriction pattern with *Bcu*I restriction enzyme. Only one or two different PFPs of this microorganism were obtained from the same animal (Table 2).

However, a wide diversity of PFPs was obtained from animals with organic dissemination of *Streptococcus* species. The PFGE analysis of 18 *S. porcinus* isolates obtained from 5 animals showed nine different PFPs (9/18; GD 0.5) (Table 2). Undistinguishable PFPs were only detected in one pig (1/5; 20%) (Table 2). The 17 *S. dysgalactiae* spp. *equisimilis* isolates recovered from 6 animals displayed eight different PFGE patterns (8/17, GD 47.06) with two animals showing the same clone in all organs (2/6; 33.34%) (Table 2).

Although our experimental design was not set up to analyse the diversity of the recovered isolates between different farms and the sample size was limited, the PFGE analysis showed some information of interest. Thus, isolates with high genetic similitude of *T. pyogenes* were detected from animals belonging either to the same farm or to different swine herds (farms 5, 6, 7 and 12). However, a high genetic heterogeneity was observed within the isolates of *S. porcinus* and *S. dysgalactiae* spp. *equisimilis*, with genetically different isolates circulating intra-herd and inter-herds (Table 2).

## Discussion

Tuberculosis like lesions (TBL) remains as one of the main causes of condemnation in swine reared in outdoor systems, producing significant economic losses [7, 17]. In a retrospective study carried out in southern Spain (2011 to 2016) 85% of totally condemned pig carcasses were related to generalised TBL [19]. According to the heterogeneity in pathology, microbiology and immunological features of TBL, the present study was designed to determine the organic distribution of MTC, *T. pyogenes* and streptococci as the main etiologic agents involved in TBL in free-range pigs as well as to characterise histological lesions of generalised TBL and the molecular diversity of *T. pyogenes* and streptococci species.

Microscopically, TBL are characterised as pyogranulomas and granulomas at different evolutionary stages (stages I to IV) in lymph nodes and other organic locations. Although different studies suggest that TBL are frequently limited to head lymph nodes [23], different body locations such as other lymph nodes or thoracic or abdominal organs can be also affected in pigs [15]. In our study, microscopic lesions were mainly observed in SLN and GHLN, with only occasional involvement of SILN and PLN, and in a lesser extent in internal organs, such as lungs, liver and spleen. The fact that stage III and stage IV granulomas were detected both in SLN and GHLN support the hypothesis that both the respiratory and digestive routes of infection play an important role in pigs, as previously suggested [23, 26]. Early stage granulomas (stage I and stage II) were mainly observed in lungs, liver and spleen, which suggests that the infection in these organs was more recent and probably associated to the dissemination from a primary focus which might be potentially present in SLN or GHLN.

Granulomas with different evolutionary stages were observed within the same organ. These findings can be explained because tuberculous lesions may change over time coinciding with periods of exacerbation or remission of the disease [18] and may be related to the balance of the local immune response within each granuloma, with slight differences in inflammatory pathways contributing to diverse granuloma architectures and functions, which may have different consequences for bacterial control [22]. Our results highlight the importance of evaluating histological characteristics of granulomas to better understand the pathogenesis of the disease as well as in the monitoring of control measures.

MTC was detected in 31 (83.78%) out of 37 animals and 90 (90/262) samples. These results were expected according to the convenient selection of farms with previous history of TBL and raised under outdoor systems, sharing resources with other domestic and wild species, such as bovine and caprine species, wild boar and wild ruminants, that play a role in the direct or indirect transmission of the disease to this species [26]. In the six MTC^−^ animals detected in our study stage IV granulomas were the most common ones in the lymph nodes; however, stage I and stage II granulomas were also observed. The negative results obtained in our study may be, at least in part, justified by the difficult detection of MTC DNA from deeply necrotic and mineralised lesions or by splitting up the lesions to be included in the histopathological study as well as in qPCR analysis during the sampling.

In MTC^+^ animals, mycobacteria were detected most frequently in SLN (24/31; 77.42%) and GHLN (19/31; 61.29%). It has been suggested that the digestive tract is an important route of transmission of tuberculosis in pigs due to the consumption of contaminated feed, which could explain the high frequency of detection in this organic location [1, 10]. It is interesting to highlight that examination of both SLN and GHLN allowed the detection of 93.55% of the MTC^+^ animals. Therefore, both lymph nodes should be included in the sampling in epidemiological surveillance programs to improve the sensibility in the identification of positive animals. Furthermore, MTC was also detected in other organs, such as spleen (9/31), liver (8/31), lungs (8/31) and tonsils (4/31), as evidenced in wild boar [3, 23], with the potential risk of excretion by numerous routes (nasal secretions, oral, faeces) and transmission to other animals by direct or indirect contact as well as to the environment. Further studies are encouraged to determine the epidemiological role of this species in the maintenance of the disease in outdoor systems.

In addition to MTC, we found a broad range of microorganisms in different organic locations, with *Trueperella pyogenes*, *S. dysgalactiae* spp. *equisimilis* and *S. porcinus* as the main non-tuberculous pathogens detected, alone or in combination with MTC. A wide distribution of these microorganisms in different body compartments was also observed; *T. pyogenes* was frequently isolated from TBL in the liver (23.53%) and lymph nodes (18.92% SLN and 19.44% SILN, respectively); *S. porcinus* was detected mainly in tonsils (42.86%), lungs (13.51%) and SLN (10.81%); and *S. dysgalactiae* spp. *equisimilis* in SLN (13.51%) and lungs (13.51%). These species have already been associated with a high rate of whole carcass condemnation due to generalised lymphadenitis in free-range pigs [7, 25], emphasizing the importance of implementing control strategies against these microorganisms to reduce the impact of carcass condemnation at the slaughterhouse.

Both *T. pyogenes* and *Streptococcus* spp. are considered ubiquitous and opportunistic pathogens that cause different clinical conditions in pigs. Pigs are usually healthy carriers of these microorganisms in the skin, tonsils or respiratory, genitourinary and gastrointestinal tracts, but in addition these microorganisms can also be found in the environment, which favours continuous infections and reinfections of the animals [14]. Therefore, we decided to determine the genetic similarity of the isolates obtained at different organic locations from each animal using PFGE techniques [31]. In our study, *T. pyogenes* characterisation was performed using 6 different restriction enzymes and different incubation times, including *Sfi*I, *Sma*I, *Bsp*120I, *Xba*I, *Xho*I and *Bcu*I (data not shown). According to our preliminary study, the proposed PFGE protocol, based on restriction with *Bcu*I (10 IU, 4 h at 37 °C), is an adequate method for the genetic characterisation of *T. pyogenes*, which allowed obtaining a pattern of 15 to 18 bands well differentiated in the gel.

Four different PFGE patterns (A, B-B1, C, D-D1) at an 85% of genetic similarity were obtained from *T. pyogenes* isolates (GD 0.13). These results show the important role of this microorganism as etiological agent of porcine lymphadenitis and open the door to further studies to elucidate the pathogenesis and control measures of interest, such as vaccine-based strategies, against this disease.

However, a wide diversity of PFGE patterns were observed for *Streptococcus* species, *S. porcinus* isolates, with nine different PFGE patterns (GD 0.5), and *S. dysgalactiae* spp. *equisimilis* isolates, with eight different PFGE patterns (GD 0.47). These results are in agreement with previous studies of genetic diversity of streptococci from animals including *S. porcinus* [11], *S. dysgalactiae* spp. *equisimilis* [9] and *S. suis* [20, 27] and evidence that TBL can be produced by different isolates, which should be taken into account when applying control measures against the disease, based on management and biosecurity measurement.

This information will allow gaining knowledge of interest to decipher the pathogenesis of TBL in pigs as well as identifying target organs for the diagnosis of the main pathogens involved in this process to avoid misdiagnosis and the implementation of holistic control measures.

## Conclusions

Results of this study show that the SLN and GHLN are the most frequently organs affected from MTC, and they can be selected for the diagnosis in the surveillance programs of tuberculosis in pigs. Furthermore, other pathogens, such as *T. pyogenes* and streptococci, can be involved in disseminated infections, with and without mycobacterial involvement. The high genetic similarity observed in *T. pyogenes* isolates obtained from generalised TBL in this study, point to this pathogen as a key microorganism in porcine lymphadenitis and requires the adoption of specific control strategies. On the other hand, different isolates of *Streptococcus* spp. were detected due to opportunistic character of this species. Our results highlight the importance of establishing an adequate diagnosis to adopt the most appropriate control measures, such as those based on vaccination and biosecurity strategies.

## Material and methods

### Experimental design and sampling

A total of 37 free-range pigs with whole carcass condemnation due to generalised TBL according to the European Regulation for meat inspection (Regulation 2004/854/EC Regulation (EC) No 854/2004 of the European Parliament and of the Council of 29 April 2004 laying down specific rules for the organisation of official controls on products of animal origin intended for human consumption. OJ L 139, 30.4.2004, p. 206–320) were selected and sampled at slaughterhouse. All animals were apparently healthy free-range pigs over 14-month-old raised in extensive systems from 16 farms located in southern Spain. To choose these animals, farms were conveniently selected according to two criteria: (1) farms with a previous history of condemnation due to TBL during the last 5 years and (2) farms with fattening pigs which can be followed at the slaughterhouse where the sampling was accomplished. Pigs from the selected farms slaughtered in two consecutive campaigns were evaluated by official meat inspectors and all condemned animals due to generalised TBL were sampled and included in the study. A minimum of one and a maximum of six pigs per farm were sampled and after routine meat inspection procedures, a total of 262 samples from 37 animals were obtained and distributed as follow: submandibular (SLN, 37), superficial inguinal (SILN, 37), gastrohepatic (GHLN, 36), and popliteal (PLN, 36) lymph nodes, lungs (37), liver (34), spleen (24) and tonsils (21). Lung, liver and spleen samples were only collected when compatible gross lesions were observed. Scattered samples from the same animal were not occasionally available due to sampling by the official veterinary services for routine diagnosis. To avoid cross contamination, different sets of sterile instruments and vials were used to collect and transport samples from each animal.

Whenever possible, one well-defined lesion was selected from each organ and was divided into two portions: one portion was subjected to histopathological analysis and the other one was immediately submitted to bacteriology and frozen at − 20 °C to perform qPCR assays. However, when small-sized disseminated (miliar) lesions were observed, similar in gross appearance and close lesions were selected and submitted to each analysis. Different sets of sterile instruments and vials were used to avoid cross contamination.

### Histopathological analysis

Tissue samples were fixed in 10% neutral buffered formaldehyde, routinely processed, and embedded in paraffin blocks. Four μm sections were stained with haematoxylin and eosin and examined by light microscopy. Each sample was classified according to the identification of specific structures as described by Cardoso-Toset et al. [7]. Thus, granulomas were classified into four stages (I-IV) based on the pathological characterisation of TB granulomas [7, 32].

### TB diagnosis

Presence of *MTC* and *MAC* was tested by an in-house duplex qPCR [6]. Briefly, fat and connective tissue were removed from samples and up to 2 g of tissue were homogenised in a stomacher with 10 ml of sterile distilled water for 2 min. The obtained solution was centrifuged for 10 min at 1400 *g* resulting in a pellet for each sample. Genomic DNA was extracted from 25 mg of tissue homogenate using NucleoSpin® Tissue DNA isolation kit (Macherey-Nagel GmbH, Düren, Germany) according to the manufacturer’s instructions.

qPCR reactions were run in duplicate in a Agilent Technologies Mx3000P thermocycler under the following conditions: initial denaturation at 95 °C for 10 min, 40 cycles of amplification consisting of denaturation at 95 °C for 30 s, primer annealing at 65 °C for 30 s, and extension at 72 °C for 30 s. DNA from *M. bovis* and *M. avium* isolates and non-template controls were included in each assay and used as positive and negative controls, respectively.

### Bacterial isolation

Samples were plated on Blood Agar Base and Columbia Blood Agar Base with nalidixic acid and colistin sulfate (Oxoid ltd., Hampshire, UK), supplemented with 5% sterile defibrinated sheep blood and incubated both in aerobic and microaerophilic (5% CO_2_) conditions at 37 °C for 48 h. Colonies were selected and identified as previously described [7]. Further biochemical identification was performed using commercial identification galleries (API®Coryne and API®20Strep, bioMérieux, Marcy-l’Etoile, France) according to manufacturer’s instructions. Isolates were identified as a species only if identification scores in the multi-substrate identification systems were excellent, very good or good (90.0–99.9% ID); otherwise, identification was made only at the genus level (*spp*.). Latex agglutination test (Streptococcal grouping kit, Oxoid ltd, Hampshire, UK) and Christie Atkins Munch-Petersen test (CAMP test) were used for identification according to previous reports [30]. Pure cultures of each isolate were stored at − 70 °C.

### Pulsed-field gel electrophoresis (PFGE) analysis

The genetic similitude of *T. pyogenes* isolates was determined by genomic DNA digestion with *Bcu*I. Briefly, *T. pyogenes* isolates were grown on Blood agar with 5% defibrinated sheep blood (Oxoid ltd) an incubated under microaerophilic (5% CO_2_) conditions at 37 °C for 24–48 h. Isolates were harvested for preparing agarose plugs as described previously by Vela et al. [31]. DNA plugs were equilibrated in restriction buffer for 30 min at 37 °C followed by digestion for 4 h at 37 °C in 150 μl of reaction mixture containing 10 U *Bcu*I (Thermo Fischer Scientific Inc., USA). Macrorestriction fragments were separated on a 1% agarose gel at 14 °C with 0.5X TBE (Tris–Borate–EDTA) buffer. Electrophoresis was done using a constant voltage of 6 V/cm for 24 h on a CHEF DR-III electrophoresis system (Bio-Rad Laboratories; Hercules, CA, USA). The pulse time was ramped from 0.1 to 10 s and *Salmonella* serotype Branderup strain H9812 was digested with *Xba*I and included for DNA fragment size determination [13].

The genetic typing of *Streptococcus* isolates was done by PFGE after genomic DNA digestion with *Bsp*120I and *Sma*I following the protocol described by Vela et al. [19]. All the PFGE patterns (PFPs) obtained in this study were visually examined and classified as different PFPs according to criteria of Tenover et al. [28].

## Data Availability

All datasets used in this study are available from the corresponding author on reasonable request.

## Abbreviations

DNA: Deoxyribonucleic acid
GD: Genetic diversity
GHLN: Gastrohepatic lymph node
*M. bovis*: *Mycobacterium bovis*
*M. avium*: *Mycobacterium avium*
MAC: *Mycobacterium avium* complex
MTC: *Mycobacterium tuberculosis* complex
PFGE: Pulsed-field gel electrophoresis
PFPs: PFGE patterns
PLN: Popliteal lymph node
qPCR: Quantitative polymerase chain reaction
SILN: Superficial inguinal lymph node
SLN: Submandibular lymph node
*S. dysgalactiae* spp. *equisimilis*: *Streptococcus dysgalactiae* subspecies *equisimilis*
*S. porcinus*: *Streptococcus porcinus*
TBL: Tuberculosis like lesions
*T. pyogenes*: *Trueperella pyogenes*
